# Regulation of myoglobin in hypertrophied rat cardiomyocytes in experimental pulmonary hypertension

**DOI:** 10.1007/s00424-016-1865-y

**Published:** 2016-08-30

**Authors:** E. L. Peters, C. Offringa, D. Kos, W. J. Van der Laarse, R. T. Jaspers

**Affiliations:** 1 Laboratory for Myology, Faculty of Behavioral and Movement Sciences, Department of Human Movement Sciences, MOVE Research Institute Amsterdam, Vrije Universiteit Amsterdam, van der Boechorststraat 7, 1081 BT Amsterdam, The Netherlands; 2 Department of Physiology, Institute for Cardiovascular Research, VU University Medical Center, Amsterdam, The Netherlands

**Keywords:** Myoglobin, Heart failure, Oxidative capacity, Pulmonary hypertension, Hypertrophy, Growth factor, Cardiac myocyte, Protein synthesis, Protein degradation, Mitochondrial biosynthesis

## Abstract

A major problem in chronic heart failure is the inability of hypertrophied cardiomyocytes to maintain the required power output. A Hill-type oxygen diffusion model predicts that oxygen supply is limiting in hypertrophied cardiomyocytes at maximal rates of oxygen consumption and that this limitation can be reduced by increasing the myoglobin (Mb) concentration. We explored how cardiac hypertrophy, oxidative capacity, and Mb expression in right ventricular cardiomyocytes are regulated at the transcriptional and translational levels in an early stage of experimental pulmonary hypertension, in order to identify targets to improve the oxygen supply/demand ratio. Male Wistar rats were injected with monocrotaline to induce pulmonary hypertension (PH) and right ventricular heart failure. The messenger RNA (mRNA) expression levels per nucleus of growth factors insulin-like growth factor-1Ea (IGF-1Ea) and mechano growth factor (MGF) were higher in PH than in healthy controls, consistent with a doubling in cardiomyocyte cross-sectional area (CSA). Succinate dehydrogenase (SDH) activity was unaltered, indicating that oxidative capacity per cell increased. Although the Mb protein concentration was unchanged, Mb mRNA concentration was reduced. However, total RNA per nucleus was about threefold higher in PH rats versus controls, and Mb mRNA content expressed per nucleus was similar in the two groups. The increase in oxidative capacity without an increase in oxygen supply via Mb-facilitated diffusion caused a doubling of the critical extracellular oxygen tension required to prevent hypoxia (PO_2crit_). We conclude that Mb mRNA expression is not increased during pressure overload-induced right ventricular hypertrophy and that the increase in myoglobin content per myocyte is likely due to increased translation. We conclude that increasing Mb mRNA expression may be beneficial in the treatment of experimental PH.

## Introduction

Myoglobin is an oxygen buffer and transporter and substantially contributes to mitochondrial oxygen supply, particularly at low intracellular oxygen tension (<10 mmHg) [63]. The myoglobin content is decreased in several models of chronic heart failure (CHF), including dog, turkey, and chicken models, which correlates with biochemical and physiological markers of myocardial performance [33]. A decrease in myoglobin (Mb) concentration has also been reported in rat models of pulmonary hypertension (PH) with progressive heart failure (HF) [22, 40, 53] but not with stable HF [31, 40]. Furthermore, a reduction of Mb was observed in necropsies of the right-sided myocardium of pulmonary hypertensive patients [40]. These studies suggest that Mb deficiency may be a determinant of the progression of CHF due to chronic pressure overload.

Apart from oxygen transport and buffering, Mb also facilitates intracellular fatty acid transport and regulates fatty acid metabolism [50]. This is emphasized by the observation that heart muscle in mice lacking Mb (myo^−/−^) switches towards glycolytic metabolism [10]. Mb also regulates oxygen supply and consumption by generation and/or scavenging of nitric oxide (NO) [64], which enables vasodilation [11] or reduces mitochondrial oxygen consumption via inhibition of complex I and/or complex IV [4]. This can protect the heart from oxidative stress in hypoxia [11]. In addition, Mb has-suicidal-peroxidase activity [9] and serves as an iron store [5]. A substantial proportion of CHF patients is iron deficient [52]. Also, mice lacking Mb (myo^−/−^) showed differential gene expression patterns upon induction of isoproterenol-induced heart failure, suggesting a role for Mb in adaptation to overload [30].

Chronic pressure overload induces extensive myocardial hypertrophy [6, 22, 40, 53], which reduces wall stress, but also decreases mechanical efficiency in hypertrophied rat papillary muscle [65], especially when cardiomyocyte cross-sectional area (CSA) becomes larger than approximately 400–500 μm^2^ [65]. Thus, the oxygen demand of hypertrophied myocytes increases several fold, and the extracellular oxygen tension required to prevent hypoxic cores when mitochondria are maximally activated (PO_2crit_) may become limiting [6, 40, 53, 54], also because capillary density is reduced [40, 51, 60]. Hence, a mismatch between oxygen demand and supply arises and either cardiomyocyte hypoxia develops [6, 49, 54] or metabolism must be inhibited [2], which in either case results in reduced energy for contraction and cardiac output.

CSA and oxidative capacity of a muscle are normally under tight control and show a strong inverse relationship that closely fits the Hill-type diffusion model [59]. It is therefore likely that the potential to increase CSA and VO_2max_ simultaneously is limited by oxygen diffusion. Thus, cardiomyocytes can likely sustain greater cell size, increased oxidative capacity and higher workload only when Mb concentration and/or number of capillaries per myocyte increases [6, 53, 59]. The latter does not occur within 4 weeks in our model of experimental PH [6, 40, 51], but we have previously found that a monocrotaline dose of 40 mg/kg was lethal in rats with a low myoglobin concentration in right-sided cardiomyocytes (≈0.25 mM [6]) whereas compensated hypertrophy developed when the concentration of myoglobin was high (≈0.6 mM [31, 40]). The reason why myoglobin concentrations differed between these studies is not known, but could be related to food composition or housing conditions [46]. Increasing the myoglobin concentration in skeletal muscle by iron therapy in iron-deficient PH patients has some beneficial effects [39].

The mechanisms underlying the regulation of Mb during hypoxia and increased contractile activity are not yet fully understood [21]. Contractile activity increases Ca^2+^ levels and thereby activates the calcineurin (CN)-nuclear factor of activated T cells (NFAT)/myocyte enhancer factor 2 (MEF2) pathway, which is known to stimulate Mb expression [20] as well as pathological hypertrophy [29]. Also, a progressive increase in Mb messenger RNA (mRNA) and protein has been demonstrated in rats following thyroid hormone T_3_ treatment, where Mb levels exceeded euthyroid levels [14]. Type 3 deiodinase (D3) is an inhibitor of T_3_ activity and is expressed locally in the hypertrophied heart by a hypoxia-inducible factor (HIF)-1α-dependent pathway [49]. The final outcome of these signaling pathways with respect to the Mb concentration in progressive experimental PH is a heterogeneous reduction of the myoglobin concentration in right ventricular myocytes [49].

There are several possibilities why the Mb concentration lags behind the rate of cardiomyocyte hypertrophy. First, the capacity of transcription could be the limiting factor in hypertrophied cardiomyocytes, because the volume of cytoplasm per nucleus increases twofold in 2 weeks [6]. However, Ruiter et al. [40] showed that myoglobin mRNA per nucleus increased by a similar factor in stable PH 40 days after the monocrotaline injection but not in progressive PH 35 days after the monocrotaline injection (at a similar degree of hypertrophy), causing a reduced Mb mRNA concentration in progressive PH at the time of sacrifice. Furthermore, it may be that the translation of Mb mRNA is slow or inefficient in progressive PH. It is also possible that increasing ROS production causes Mb degradation. These data suggest that Mb mRNA expression is inadequate in progressive HF but also indicate that it can be upregulated in overloaded heart muscle. Hence, the aim of this study was to explore how the Mb concentration and the oxidative capacity are regulated at an early stage of progressive PH in concordance with cell size.

We hypothesized that Mb mRNA expression does increase at an early stage of the development of progressive myocardial hypertrophy. We focused on transcriptional (mRNAs: Mb, peroxisome proliferator-activated receptor gamma coactivator 1-alpha [PGC-1α], succinate dehydrogenase [SDH], cytochrome c oxidase [COX], and vascular endothelial growth factor [VEGF]) and translational control of protein synthesis (ribosomal RNA [rRNA], insulin-like growth factor-1Ea [IGF-1Ea], and mechano growth factor [MGF]) and protein degradation (muscle RING-finger protein-1 [MuRF1], muscle atrophy F-box [Mafbx], BCL2/adenovirus E1B 19 kDa interacting protein 3 [BNIP3]) and glycolytic metabolism (glyceraldehyde 3-phosphate dehydrogenase [GAPDH]).

## Methods

### Animals and preparations

The study was approved by the Animal Experimental Committee of the Vrije Universiteit Amsterdam (Amsterdam, The Netherlands) and conformed to the guide of the Dutch Research Council for care and use of laboratory animals. Male Wistar rats (*n* = 13) obtained from Harlan (Horst, The Netherlands) were injected subcutaneously with 60 mg/kg monocrotaline (MCT) at 170–190 g body mass to induce progressive right ventricular HF. This protocol causes a reduction of cardiac output of 25 to 30 % after 3.5 to 4 weeks [16, 65]. Untreated rats (*n* = 10) were used as controls. All animals received water and standard rat chow (Teklad 2016, Envigo, UK) ad libitum. Three weeks after MCT treatment, rats were anesthetized with halothane and the hearts were rapidly excised and perfused with Tyrode solution (120 mM NaCl, 5 mM KCl, 1.2 mM MgSO_4_, 2.0 mM Na_2_HPO_4_, 27 mM NaHCO_3_, 1 mM CaCl_2_, 10 mM glucose and 20 mM 2,3-butanedione monoxime, equilibrated with 95 %/5 % O_2_/CO_2_ at pH 7.6 and 10 °C) to prevent contraction and to remove blood. Biopsies of the right ventricular wall were embedded in 15 % (*w*/*v*) gelatine in Tyrode, pH 7.5, and then frozen in liquid nitrogen. Sections of 5 μm thickness were cut and either air dried for 15 min prior to the determination of SDH activity (see below) or stored at −80 °C for later analysis of the Mb concentration.

### Succinate dehydrogenase histochemistry and determination of cross-sectional area of cardiomyocytes

SDH activity was measured in the incubation medium (37.5 mM sodium phosphate buffer, pH 7.6, 75 mM sodium succinate, 5 mM sodium azide, and 0.4 mM tetranitro blue tetrazolium) as previously described [34]. Briefly, sections were incubated in the dark for 7 min at 37 °C [6]. The spatially averaged absorbance of individual cells in each section was measured at 660 nm using a calibrated microdensitometer [22] and is expressed as the rate of staining in absorbance units per micrometer section thickness and per second incubation time (Δ*A*_660_ μm^−1^ s^−1^). SDH activity is proportional to VO_2max_ under hyperoxic conditions in vitro [6, 56]. The measurement included the determination of the CSA of the cell. Absorbance was measured in 20 myocytes, so that a reliable estimate of the mean value was obtained. NIH Image and Image J (http://rsbweb.nih.gov/ij/) were used for analysis-taking the pixel-to-aspect ratio into account.

### Myoglobin concentration

For the determination of Mb concentration, sections were first fixed in paraformaldehyde vapor and subsequently in 2.5 % glutaraldehyde solution for 10 min [53]. Sections were then incubated for 1 h in 59 ml of 50 mM TRIS/80 mM KCl buffer, pH 8.0 which contained 25 mg ortho-tolidine dissolved in 2 ml 96 % ethanol at 50 °C and 1.43 ml of 70 % tertiary-butyl-hydroperoxide **(**Fluka Chemie, Switzerland**)** [22, 53]. Absorbance was measured at 436 nm and converted to Mb concentration using gelatin sections containing known equine Mb (Sigma, The Netherlands) concentrations.

### Calculation of PO_2_crit

An estimate of the minimal extracellular oxygen tension required to prevent hypoxic cell cores when mitochondria are maximally active (PO_2_crit) of the cardiomyocytes can be calculated as follows [17, 32]:

1 $$ {PO}_{2\mathrm{crit}}=\left({VO}_{2 \max}\cdot {R}^2-4{D}_{\mathrm{M}\mathrm{b}}\cdot {MbO}_{2\mathrm{R}}\right)/4{\alpha}_{\mathrm{M}}\cdot {D}_{\mathrm{O}2} $$

where VO_2_ is the rate of oxygen consumption (mM s^−1^), *R* is the radius of the cell, *D*_Mb_ is the diffusion coefficient for Mb in the sarcoplasm, *α*_M_ is the solubility of oxygen in the muscle, and *D*_O2_ is the diffusion coefficient for oxygen in muscle tissue. Furthermore, MbO_2R_ depends on PO_2_crit, the concentration of oxygenated and deoxygenated Mb (Mb_tot_), and the half-saturation pressure of Mb (*P*_50_) as follows:

2 $$ {MbO}_{2\mathrm{R}}={PO}_{2\mathrm{crit}}\cdot {\mathrm{Mb}}_{\mathrm{tot}}/\left({PO}_{2\mathrm{crit}}+{P}_{50}\right) $$

Substitution of the latter into the first equation allowed the calculation of PO_2_crit as a function of parameters that were measured or estimated using calibrated histochemistry [53] or obtained from literature (see below).

To estimate PO_2crit_ at VO_2max_, VO_2max_ was estimated from measured SDH values based on previous observations that showed SDH activity to be proportional to VO_2max_ with a staining rate of 1 · 10^−4^ Δ*A*_660_ μm^−1^ s^−1^ corresponding to a VO_2max_ of 0.6 mM s^−1^ [6, 56]. The concentration of Mb was determined from the heart sections as described above. All other values were obtained from literature: *D*_Mb_ = 0.27 · 10^−4^ mm^2^ s^−1^ [3], *α*_m_ · DO_2_ = 2 nM mm^−2^ s^−1^ mmHg^−1^ [55], and *P*_50_ = 6.5 mmHg [8, 13, 45]. Note that this calculation provides an underestimate of PO_2crit_ because zero-order kinetics for mitochondrial oxygen consumption and equilibrium of the reaction of myoglobin with oxygen are assumed (see [41] and [7], respectively, for discussion).

### Quantitative polymerase chain reaction (qPCR)

Parts (mean mass 68.8 ± 8.24 mg) of the right ventricular free wall were weighed while frozen. Total RNA was extracted using a RiboPure kit (Applied Biosystems, Carlsbad, CA) according to the manufacturer’s instructions.

Real-time PCR was performed using a StepOne Real-Time PCR system (Applied Biosystems) to determine mRNA expression levels. From each muscle, 500 ng total RNA was reverse transcribed using an RNA-to-cDNA kit (Applied Biosystems). For each gene target, 5 μl of the reverse transcribed reaction product was amplified using Fast SYBR Green Mastermix (Applied Biosystems). The primers used are listed in Table 1.

**Table 1:** Overview of primers used for RT-PCR

Gene	Forward (5′-3′)	Reverse (3′-5′)
18S	CGAACGTCTGCCCTATCAACTT	ACCCGTGGTCACCATGGTA
Myoglobin	CCGGTCAAGTACCTGGAGTTTA	TCCCCGGAATATCTCTTCTTC
VEGF	CTGCTGTGGACTTGAGTTGG	AAGACCACACCGGAGTCTTT
IGF-1Ea	AAGCCTACAAAGTCAGCTCG	TCAAGTGTACTTCCTTCTGAGTC
MGF	CAAGACTCAGAAGTCCCAGC	AAGTGTACTTCCTTTCCTTCTC
MuRF1	TGCCCCCTTACAAAGCATCTT	CAGCATGGAGATGCAATTGC
Mafbx	TGAAGACCGGCTACTGTGGAA	CGGATCTGCCGCTCTGA
BNIP3	GTCACTTCCCAGGCCTGTCGC	TACCCAGGAGCCCTGCAGGTTCT
GAPDH	TGGCCTCCAAGGAGTAAGAAAC	GGCCTCTCTCTTGCTCTCAGTATC
PGC-1α	ATGAGAAGCGGGAGTCTGAA	GCGCTCTTCAATTGCTTTCT
SDH	CAGAGAAGGGATCTGTGGCT	TGTTGCCTCCGTTGATGTTC
COX1	TGCCAGTATTAGCAGCAGGT	GAATTGGGTCTCCACCTCCA
COX4	AGTCCAATTGTACCGCATCC	ACTCATTGGTGCCCTTGTTC

*BNIP3* BCL2/adenovirus E1B 19 kDa interacting protein 3, *COX* cytochrome c oxidase, *GAPDH* glyceraldehyde 3-phosphate dehydrogenase, *IGF* insulin-like growth factor, *Mafbx* muscle atrophy F-box, *MGF* mechano growth factor, *MuRF1* muscle RING-finger protein-1, *PGC-1α* peroxisome proliferator-activated receptor gamma coactivator 1-alpha, *SDH* succinate dehydrogenase, *VEGF* vascular endothelial growth factor

Mean cycle thresholds were converted to relative expressions by subtracting the 18S rRNA cycle threshold and determining 2^−ΔCt^. Expressions relative to 18S rRNA were multiplied by total RNA per milligram of heart tissue to obtain mRNA concentrations. By multiplying the concentration by the mean CSA of the cardiomyocytes, expression levels of the genes per nucleus were determined. This normalization is based on the observations that the number of myocyte nuclei does not change during the development of hypertrophy [54] and that myocyte length does not change [57]. In this case, the volume of cytoplasm per nucleus is proportional to myocyte CSA and thus normalization for CSA reflects changes in gene expression per nucleus. It should be noted that the expression per nucleus is therefore not an absolute value but rather a relative measure.

### Statistical analysis

Independent *t* tests were used to compare measurements from MCT-treated animals with those of the control animals. Equality of variance was tested using Levene’s test and corrected if significant. Normality was tested using the Shapiro-Wilk test. For data with a non-normal distribution, the Mann-Whitney *U* test was used. Values are given as mean ± standard error of the mean (SEM) unless stated otherwise; *p* < 0.05 was considered statistically significant.

## Results and discussion

### Effects of MCT on cardiomyocyte phenotype

**Fig. 1:**
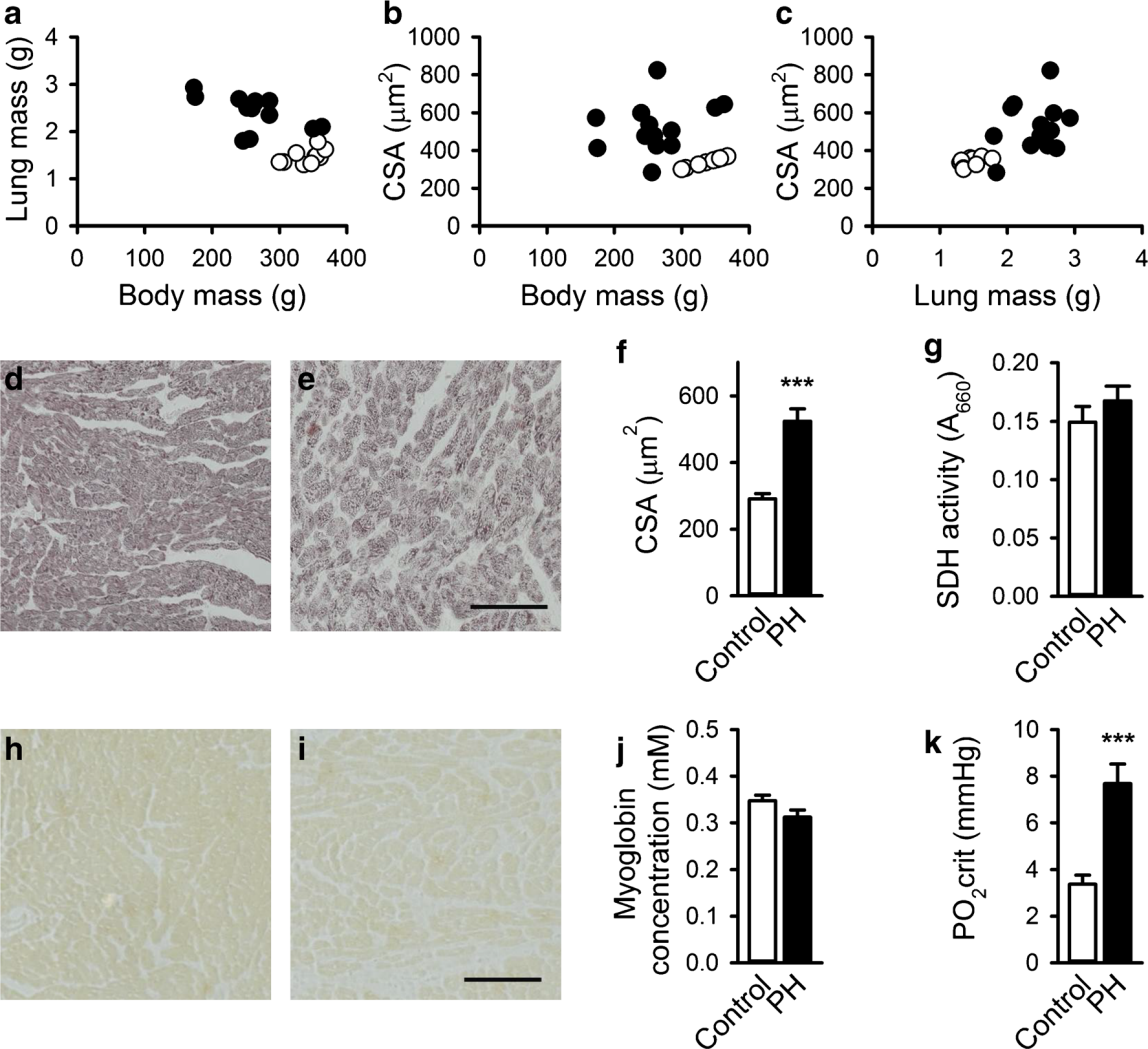
Effects of monocrotaline-induced pulmonary hypertension on phenotypic characteristics of the rats and cardiomyocytes in rat right ventricle. Lung mass (**a**) and myocyte CSA (**b**) are plotted against body mass, and myocyte CSA is plotted against lung mass (**c**). Representative examples of control and PH right ventricular cardiomyocytes stained for succinate dehydrogenase (SDH) activity (**d**, **e**) and myoglobin (**h**, **i**) are shown. MCT-induced heart failure caused hypertrophy of cardiomyocytes, as illustrated by an increase in cellular cross-sectional area (CSA) (**f**). Nevertheless, SDH activity (**g**) and myoglobin protein concentration (**h**–**j**) both remained constant. Therefore, PO_2crit_ was increased in PH (**k**). ***p < 0.001. *White bars/circles*: control group, *black bars/circles*: PH. *Scale bar* indicates 100 μm

Figure 1 shows lung mass and RV myocyte CSA against body mass, CSA against lung mass, and CSA, SDH activity, and Mb concentration both for PH rats and controls. Lung mass and CSA were higher in MCT-treated rats although body mass was lower (Fig. 1a–c) illustrating the detrimental effects of the MCT injection after 21 days.

Cardiomyocyte CSA of MCT rats thus increased 1.8-fold compared to that of controls (*p* < 0.001; Fig. 1d–f), confirming hypertrophy. Based on the hyperbolic inverse relationship between muscle fiber size and oxidative capacity [56, 59], we expected to see a decrease in oxidative capacity during hypertrophy. However, SDH activity was similar in PH rats and controls (*p* = 0.34; Fig. 1d, e, and g), indicating that oxidative capacity per unit volume of cytoplasm was retained after MCT injection. Since CSA was increased, the total oxidative capacity per cardiomyocyte increased. As this is accompanied by higher oxygen consumption per cardiomyocyte, these hypertrophied cells would require increased Mb concentrations to prevent hypoxia. However, Mb concentrations in PH and control samples were not statistically different (*p* = 0.11; Fig. 1h–j).

The increase in absolute oxidative capacity without a concomitant increase in Mb protein concentration led to a PO_2crit_ in PH (7.7 mmHg) that was over twofold greater than the PO_2crit_ (3.4 mmHg) in controls (*p* < 0.001; Fig. 1k). The increase in PO_2crit_ and the decrease in the capillary density [40, 51, 60] are likely to cause core hypoxia in cardiomyocytes at the maximum heart rate [54].

The Mb concentrations in the present study are different from those previously reported [6, 22, 53]. This variation indicates that Mb regulation in MCT-induced PH is complicated. The Mb concentration was previously shown to be decreased in experimental PH [40] after 4 weeks, suggesting that the decrease occurs during the fourth week, when the cardiomyocytes no longer increase in size [6].

### Effects of MCT on total RNA content in the right ventricle

The volume of cytoplasm each nucleus had to maintain (i.e., the myonuclear domain) increased with CSA. Thus, to maintain SDH activity and Mb concentration, either the rate of transcription/translation or both should have been enhanced and/or the half-life of Mb should have increased. Because 80–85 % of all RNA within muscle cells consists of rRNA [28], we first assessed total RNA content per milligram heart tissue, as a measure of translational capacity.

**Fig. 2:**
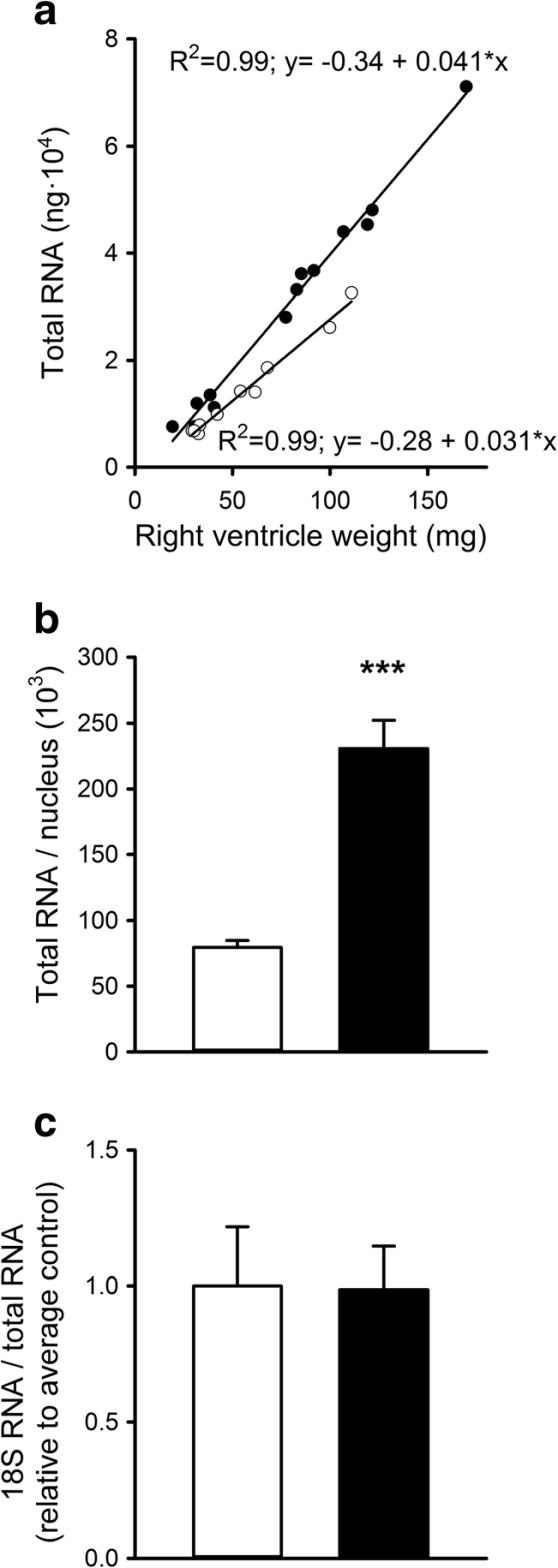
Effects of monocrotaline-induced pulmonary hypertension on total RNA content and 18S rRNA in rat right ventricle. The amount of total RNA was proportional to the weight of the right ventricle, although the relationship is different for PH rats versus controls (**a**). The mean amount of total RNA per nucleus was more than twofold greater for the PH group compared to controls (**b**). Nevertheless, the expression of 18S rRNA relative to total RNA was similar in both groups (**c**). ***p < 0.001, **p < 0.01. *White bars/circles*: control, *black bars/circles*: PH

Total RNA was proportional to wet weight (Fig. 2a; *p* < 0.001). However, the relationship differed between PH rats and controls, indicating that rats with PH had 32 % higher total RNA levels per milligram muscle tissue (*p* = 0.001). Total RNA per nucleus was 2.7 times higher in PH rats compared to controls (*p* < 0.001; Fig. 2b). Expression levels of 18S rRNA relative to total RNA were similar (*p* = 0.96; Fig. 2c). On the basis of these results, we conclude that rRNA content was proportional to the increase in total RNA, reflecting a higher overall translational capacity in PH cardiomyocytes. Hence, it is unlikely that a limitation in the translational capacity impaired the increase in Mb protein expression.

The increase in total RNA and the absolute increase of 18S rRNA expression levels in PH rats also indicate that mRNA expression levels normalized to 18S rRNA lead to an underestimation of the mRNA expression levels in PH rats compared to controls. Therefore, we normalized subsequent mRNA expression levels both as cardiac tissue mRNA concentration and as amount per nucleus.

### Effects of MCT on transcription of regulators of protein synthesis and degradation

**Fig. 3:**
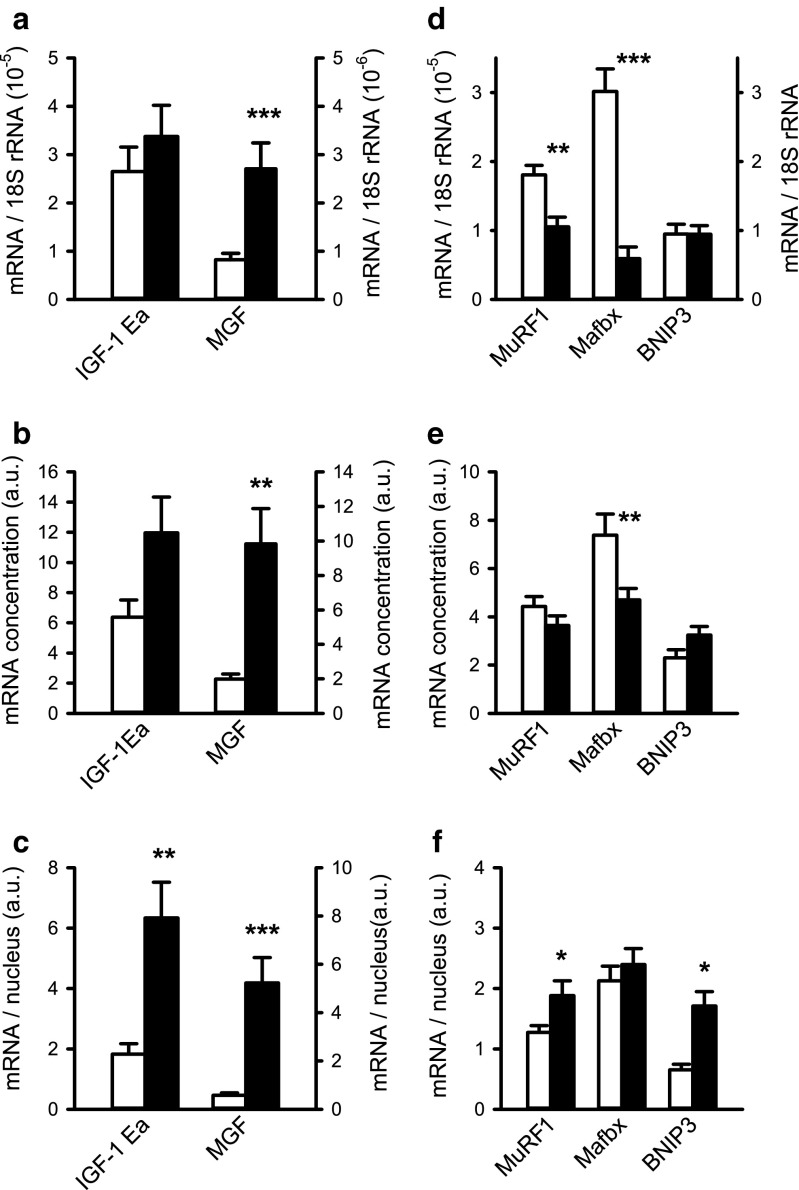
Effects of monocrotaline-induced pulmonary hypertension in rat on expression of regulators of protein synthesis and degradation in the right ventricle. Expression levels of insulin-like growth factor (IGF)-1Ea and mechano growth factor (MGF) mRNA are presented relative to 18S (**a**), as concentration per milligram heart tissue (**b**) or per nucleus (**c**). Expression levels of muscle RING-finger protein-1 (MuRF1), muscle atrophy F-box (Mafbx), and BCL2/adenovirus E1B 19 kDa interacting protein 3 (BNIP3) were analyzed as markers of degradation and shown relative to 18S (**d**), as concentration (**e**) and per nucleus (**f**). Note that the right axis in **d** applies only to BNIP3 expression levels. ***p < 0.001, **p < 0.01, *p < 0.05. *White bars*: control, *black bars*: PH

We studied mRNA expression levels of several factors related to protein synthesis and degradation as shown in Fig. 3.

In order to explain the lack of increase in Mb concentrations, we considered IGF-1Ea and its splice variant MGF, which are known to activate both the rate of transcription and translation [15] and repress several mediators of degradation [43]. In response to exercise or overload, IGF-1 acts as an autocrine/paracrine factor to induce hypertrophy in left ventricular myocardium of rats [42] and humans [47]. It has been shown that physiologic and pathologic cardiac hypertrophy are mediated by different pathways whereby IGF-1 is essential for physiologic hypertrophy and acts via the phosphatidylinositol 3-kinase (PI3K)-Akt-mammalian target of rapamycin (mTOR) pathway whereas pathologic hypertrophy is mediated by the Ca^2+^-CN-NFAT pathway (see [61] for review).

Relative to 18S rRNA expression levels and expressed as concentration (i.e., per milligram tissue), IGF-1Ea expression levels did not differ significantly between the two groups (*p* = 0.41, *p* = 0.07; Fig. 3a, b). However, expression levels of mRNA per nucleus increased 3.5-fold in PH rats compared to controls (*p* < 0.01; Fig. 3c). MGF expression levels were also increased, irrespective of whether they were expressed relative to 18S (*p* < 0.001), concentration (*p* < 0.01), or per nucleus (*p* < 0.001; Fig. 3a–c). In addition to the activation of mRNA transcription and translation, MGF has been shown to preserve cardiac function by inhibiting apoptotic pathways in the myocardium and preventing pathologic cardiac hypertrophy [27].

As expression of IGF-1Ea and MGF mRNA per nucleus were both increased, it is conceivable that the rates of transcription and translation were increased. To investigate this further, we assessed several markers of protein degradation. MuRF1 and Mafbx are known to regulate contractile protein degradation, thereby preventing massive hypertrophy in skeletal and cardiac muscle cells [25, 62]. Furthermore, BNIP3 induces mitochondrial dysfunction and autophagy [36] and apoptosis under hypoxic conditions [38]. Since SDH protein expression was unexpectedly increased, we also studied BNIP3 mRNA expression levels.

The expression levels of MuRF1, Mafbx, and BNIP3 were lower or unaltered in PH rats versus controls when expressed relative to 18S rRNA (*p* < 0.01, *p* < 0.001, and *p* = 0.99, respectively; Fig. 3d), whereas an increase was shown in the expression per nucleus for MuRF1 and BNIP3 (both *p* < 0.05) by 1.5- and 2.6-fold, respectively (Fig. 3f). This appeared to be sufficient to keep the concentration constant, whereas the concentration of Mafbx was rather decreased (*p* < 0.01) due to the constant expression per nucleus (Fig. 3e). Together, these results show a clear elevation in the mRNA concentration of growth factors involved in protein synthesis. By contrast, the concentration of E3 ligases was lower or remained constant. The increase in translational machinery and signaling was apparently sufficient to maintain but not to increase the Mb concentration.

### Effects of MCT on transcriptional expression of metabolic enzymes

**Fig. 4:**
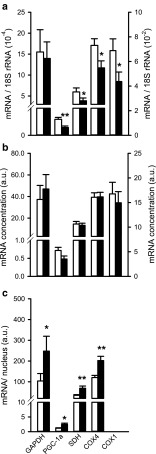
Effects of monocrotaline-induced pulmonary hypertension on expression of metabolic markers in rat right ventricle. Expression levels of glyceraldehyde 3-phosphate dehydrogenase (GAPDH), peroxisome proliferator-activated receptor gamma coactivator 1-alpha (PGC-1α), succinate dehydrogenase (SDH), cytochrome c oxidase (COX) 4, and COX1 are shown relative to 18S (**a**), as concentration (**b**) and per nucleus (**c**). Note that the right axis in a and b only applies to COX1. COX1 expression levels per nucleus are not presented because this subunit is encoded by the mitochondrial DNA. ***p < 0.001, **p < 0.01, *p < 0.05. *White bars*: control, *black bars*: PH

GAPDH catalyzes the conversion of glyceraldehyde 3-phosphate to d-glycerate 1,3-bisphosphate and is a marker of glycolytic metabolism. Expression levels of GAPDH did not differ between the two groups when expressed relative to 18S rRNA or as mRNA concentration (Fig. 4a, b). When considered per nucleus, GAPDH mRNA expression levels were 2.5-fold higher in PH rats compared to control (*p* < 0.05; Fig. 4c). Because the mRNA concentration does not decrease and the rRNA increases, this result suggests an increase of the glycolytic capacity in MCT-induced PH. A shift towards glycolytic metabolism was also observed in myo^−/−^ mice suggesting compensation for the lack of Mb [10]. However, Mb concentrations in the present study remained constant and thus do not explain the supposed increase in glycolytic capacity. Alternatively, a shift towards glycolytic metabolism that was associated with a transition towards a decompensated state in PH [51] may have accounted for the increased glycolytic capacity as shown here. Regardless of the underlying mechanism, this shift may reflect one way to lower oxygen utilization of the cardiac myocytes.

We investigated whether SDH mRNA expression levels were increased. Expressed relative to 18S rRNA, we observed a decrease in PH rats compared to controls (*p* < 0.05; Fig. 4a) while mRNA concentration remained constant (Fig. 4b). By contrast, the expression per nucleus was almost twice as high in PH rats versus controls (*p* < 0.01; Fig. 4c). Because cardiomyocyte CSA and SDH activity both increased almost twofold in PH rats versus controls, it can be concluded that SDH mRNA expression was sufficient and in line with the increase in SDH activity per myocyte.

SDH and COX activities have been shown to be proportional during the development of MCT-induced PH [31]. To confirm that SDH activity reflected the oxidative capacity, we also measured mRNA expression levels of both COX1 and COX4, subunits of cytochrome c oxidase. Although expression levels of both were decreased relative to 18S (*p* < 0.05; Fig. 4a), the mRNA concentrations remained constant (Fig. 4b). The expression of COX4 mRNA per nucleus was increased almost twofold, in line with the SDH activity (Fig. 4c). Expression per nucleus is not shown for COX1 because this subunit is encoded by the mitochondrial DNA [12]. However, the observed increase in COX4 expression per nucleus, together with the constant COX1 mRNA concentration despite the increase in cell size, indicates that expression levels of both subunits were proportional to SDH activity and were not limiting the increased oxidative capacity. Therefore, SDH activity seems an appropriate estimate of VO_2max_ used to estimate PO_2_crit.

We also investigated how SDH activity was maintained despite substantial hypertrophy. PGC-1α is known to be the master regulator of mitochondrial biosynthesis [23, 35]. Following hypoxia, its expression is increased or PGC-1α is activated because of an increase in ROS production, p38 mitogen-activated protein kinase (MAPK) and AMP-activated protein kinase (AMPK) levels [18, 48]. However, although we show that expression levels relative to that of 18S rRNA were lower (*p* < 0.01; Fig. 4a), the expression per nucleus was over twofold higher in PH rats versus controls (Fig. 4c), and there was no difference in PGC-1α mRNA concentrations between the two groups (Fig. 4c).

We conclude that the upregulation of PGC-1α per nucleus is probably the reason why the oxidative capacity was maintained. We observed that cardiomyocyte hypertrophy with maintained SDH activity requires an increase in interstitial PO_2crit_. This implies that in order to make use of all mitochondrial enzyme activity, oxygen supply to the cardiomyocytes needs to be increased.

### Effects of MCT on transcriptional regulation of proteins involved in oxygen supply or the regulation thereof

**Fig. 5:**
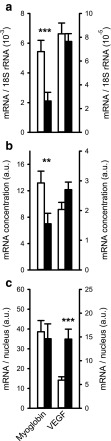
Effects of monocrotaline-induced pulmonary hypertension on mRNA expression levels of myoglobin and vascular endothelial growth factor in rat right ventricle. Expression levels of myoglobin (Mb) and vascular endothelial growth factor (VEGF) are presented for both the PH group and controls relative to 18S (**a**), as concentration (**b**) or per nucleus (**c**). ***p < 0.001, **p < 0.01, *p < 0.05. *White bars*: control, *black bars*: PH

To explain the lack of increase in Mb concentration, we assessed both Mb and VEGF mRNA expression levels, as these are indicative of changes in oxygen supply. In addition to its role in angiogenesis [24], it is suggested that VEGF can directly stimulate Mb mRNA transcription via a currently unknown mechanism [58]. An increase of VEGF expression could be either the result of enhanced expression or activation of PGC-1α [1] or resulting from stabilization of HIF-1α [48]. It is known that HIF-1α promotes VEGF-induced angiogenesis under hypoxic conditions [44]. Although we did not measure HIF-1α, it is reported to accumulate consistently in PH [6, 37, 49, 51, 54]. No differences in VEGF mRNA expression levels relative to 18S rRNA were observed (Fig. 5a), and although the expression levels per nucleus were increased in PH rats compared to controls (*p* < 0.001; Fig. 5c), there was no difference between the groups in the VEGF mRNA concentration (Fig. 5b). These findings are consistent with previous reports that VEGF protein expression remains constant both in stable and progressive HF in rats after 3 weeks of right ventricular overload [40].

Although a decrease was seen in the expression relative to 18S RNA (*p* < 0.001) and mRNA concentration (*p* < 0.01; Fig. 5a, b), the expression of Mb mRNA per nucleus remained constant in PH rats (Fig. 5c). In order to retain a stable Mb protein concentration with the increased cell size, we had expected to find a higher expression of Mb mRNA per nucleus. Our data suggest that the Mb protein concentration was maintained due to an increase in the rate of translation, rather than by an increased rate of transcription. It should be noted that we did not correct for an increase in interstitial space from 11 % in controls to 17 % in PH rats [40]. However, such a correction would only slightly increase the calculated concentration of Mb mRNA per milligram right ventricle tissue by 12–21 %, but expression levels per nucleus would remain unaltered. This extends results of Ruiter et al. [40] who showed that myoglobin mRNA was also not upregulated at later stages of progressive PH, whereas it was upregulated in compensated PH. Furthermore, after 2 weeks of isoproterenol-induced cardiac hypertrophy, Mb mRNA expression was shown to be constant [30]. However, this was expressed relative to a certain amount of RNA. Since we have shown here that total RNA increased in MCT-induced cardiac hypertrophy, this may also be the case for isoproterenol-induced cardiac hypertrophy and would increase Mb mRNA concentrations in diseased mice. Further research is needed to reveal whether Mb mRNA is only upregulated at even earlier onset of heart failure or whether Mb mRNA expression levels are differentially altered in the different models. Thus, our hypothesis is rejected and the question remains why Mb mRNA expression was not upregulated at an early stage of progressive PH. One explanation for this surprising result is that oxidative metabolism was inhibited in progressive PH [2], thereby preventing the hypoxia stimulus required for myoglobin expression, while myocytes are able to adapt to hypoxia by increasing myoglobin expression in compensated PH.

Previous studies have demonstrated regulation of Mb via Ca^2+^-CN-NFAT/MEF2 pathways [29] indicating that contractile activity may contribute to the regulation of Mb expression. Furthermore, it has been demonstrated that hypoxia in combination with contractile activity enhances Mb expression in C_2_C_12_ myotubes, mouse skeletal [20] and heart [26] muscle, and zebrafish high oxidative muscles [19]. However, this was not the case in our MCT-induced overload of the cardiomyocytes of the right ventricle, despite the fact that increased power output and reduced oxygen tension (judging from increased HIF-1α expression [6, 37, 49, 51, 54]) were likely present. However, Mb expression was increased following lipid supplementation in hypoxic C_2_C_12_ cells and rat soleus muscle, independent of CN signaling, suggesting that other pathways for Mb expression do exist [46]. Furthermore, as mentioned before, iron supplementation [39] and treatment with thyroid hormone [14, 22] both successfully increased Mb expression in PH patients and rat, respectively, and thus may serve as required additional stimuli.

In conclusion, this study shows that Mb mRNA expression was not sufficient to increase Mb protein concentrations even at an early stage of progressive PH. Upregulating Mb mRNA expression, e.g., by supplementation of iron [39] and fatty acid [46] and/or stimulation of the thyroid hormone receptor [14], is therefore a promising therapeutic strategy. Further research should reveal the optimal combination of hypoxia, load, and dietary status to increase Mb mRNA and protein levels in chronic heart failure.
